# Family-Based Intensive Trauma Treatment for Youth with PTS Symptoms After Family Violence: A Qualitative Study of Youths’ Perspectives on Treatment Outcomes, Key Components, and Barriers and Facilitators for Treatment Success

**DOI:** 10.1007/s40653-026-00904-4

**Published:** 2026-05-08

**Authors:** Valerie Fictorie, Caroline Jonkman, Margreet Visser, Majone Steketee, Carlo Schuengel

**Affiliations:** 1 Section of Clinical Child and Family Studies, Vrije Universiteit Amsterdam, Amsterdam, Netherlands; 2 Children’s Trauma Center, Kenter Jeugdhulp, Haarlem, Netherlands; 3 Department of Psychology, Education and Child Studies, Erasmus University Rotterdam, Rotterdam, Netherlands; 4 Verwey Jonker Institute, Utrecht, Netherlands

## Abstract

Family violence affects the lives of youth and their families. Treatment offers an opportunity to reduce the possible long-term negative consequences of family violence. Efforts to further develop treatments by including family relationships and condensing treatment into a high-intensive format require reflection from the perspective of youth themselves. In this study, youth in an intensive trauma treatment trial with or without a family based component, were interviewed afterwards. They reported improvements for themselves. Those who received treatment with the family component also reported improving parental and family outcomes. Youth identified as key elements the combination and variety of trauma-focused components and therapist rotation. They perceived that the family-based component created a supportive context, addressing family safety and parent mental health. Youth who did not receive the family-based component had mixed assumptions toward participation of family members. Several facilitators and barriers for treatment success were identified. Findings highlight the importance of including the voices of youth when trying to understand changes within the therapeutic process. Furthermore, youths’ preferences for treatment and their considerations on inclusion of family members need to be attended to.

## Introduction

### Background

Family violence affects the lives of many families. Globally, around one in six people report having experienced physical family violence in their childhood, as a victim or witness (Whitten et al., 2024). A meta-analysis of 95 studies with 19,389 participants in total revealed that after interpersonal trauma, such as family violence, approximately one-third of youth (Visser et al., 2025) report posttraumatic stress (PTS) symptoms. A British study showed that more than three-quarters of women, seeking help after family violence, reported PTS symptoms (Ferrari et al., 2016). Furthermore, parents report elevated levels of parenting stress (Sypher et al., 2022) and show more negative parenting, increasing the risk of negative parent-child interaction (Kopystynska et al., 2022). Given this significant impact of family violence during a critical phase of youth development, effective interventions are crucial not only for youth to recover from the psychological impact that otherwise may have long-term consequences (Felitti et al., 2019), but also to restore parent-child relationships. In evaluating interventions, involving parents and with an intensive focus, it is important to include the perspectives of youth themselves. Engagement of youth can enhance study quality (Head, 2011), such as more honest findings (Racine et al., 2023), and increase insight in outcomes of interventions that are important to youth themselves (Cuijpers, 2019). Therefore, the present study explores the perspectives of youth on intensive and family-based trauma treatment, designed for youth exposed to family violence.

### Treatment for Youth Exposed to Family Violence

Current treatments for youth exposed to family violence may fall short. First, despite the impact of family violence at both the individual and the family level, interventions for family violence (Rizo et al., 2011) and trauma-focused therapies, that are effective in reducing PTS symptoms (Hoogsteder et al., 2022), do not always focus on both youth and parents. Second, the number of randomized controlled trials that specifically focus on family violence when testing the effectiveness of trauma-focused cognitive behavioral therapy (TF-CBT) is low and dropout rates for this group are higher than found in studies on TF-CBT after other forms of trauma (Cox et al., 2025; Simmons et al., 2021). Third, in a qualitative study in Sweden, youth who participated in trauma-focused treatment after family violence reported that family safety was seldom addressed by their therapist, although the violence at home continued during the treatment period (Onsjö et al., 2022). These studies show the limitations of current treatments and emphasize that treatments may be improved by increasing tolerability of treatments and by taking into account the perspectives of youth themselves on the outcomes that matter to them in addition to a sole focus on symptom reduction (Cuijpers, 2019). Their perspectives can provide valuable insights into how they perceive and explain change in psychotherapy (Wampold, 2015).

### Family-Based Component

Pernebo and Almqvist (2016) recommended that, based on their qualitative study with youth exposed to family violence, treatment should focus on both trauma processing for the child and include treatment for the parent, focusing on parental distress, parenting skills, and parental availability. Furthermore, a review on interventions for families exposed to family violence suggested that interventions that include joint sessions next to a separate child and parent component yielded the most positive effect for parents and children (Anderson et al., 2018). Two systematic reviews on interventions that included a parent component showed effects on decreasing parenting stress (Rizo et al., 2011) and negative parenting, but only a small number of studies included these variables as an outcome measure (Lindstrom Johnson et al., 2018). Another systematic review focusing on parenting programs for mothers exposed to family violence (Austin et al., 2019) aimed to identify effective intervention components. However, conclusions could not be drawn due to the wide heterogeneity of interventions and the lack of clear intervention descriptions. Also, reporting on family safety as an outcome measure was often lacking. A meta-analysis that included family safety as an outcome measure (Hackett et al., 2016) showed that interventions after family violence that focused on both mothers and children resulted in medium-to large effects on family relationships and violence incidents. Although these findings provide promising evidence for the added value of parenting interventions, it remains unclear which specific components are most effective. Nonetheless, the results shed light on the parental factors most commonly addressed in interventions for family violence. Rizo et al. (2011) outlined a set of overarching treatment goals that closely align with the treatment elements identified in the other reviews. These treatment goals/components include addressing parental stress, enhancing coping skills, safety planning, strengthening the parent–child relationship, and increasing knowledge about family violence.

### Format of the Treatment

Besides possibly improving treatment outcomes by adding parental components to trauma treatment, it is also necessary to improve adherence and focus on timely recovery. To promote adherence and accelerate recovery, there is a recent trend to intensify trauma treatment (Oprel et al., 2021; Van Woudenberg et al., 2018). Some uncontrolled trials working with youth suggest that intensive trauma treatment may be effective (Rentinck et al., 2025) and increase adolescents’ feelings of accomplishment (van Ee et al., 2024). In intensive trauma treatment, the trauma-focused components are offered by a group of therapists, described as ‘therapist rotation’. This means, for example, that the EMDR sessions are provided by multiple therapists that follow one another in the treatment. Van Minnen et al. (2018), interviewing participants of an intensive trauma treatment, reported that 62% of participants were positive about their contacts with the rotating therapists from the team. Therapists in intensive trauma treatment reported less hesitation to initiate trauma treatment, even in cases where symptoms were severe, and expected that intensive treatment may make trauma treatment more feasible for populations with complex needs (Vaage-Kowalzik et al., 2025). This is an important finding, as in previous studies complex groups were sometimes excluded, such as youth with an unsafe home situation (Tijsseling et al., 2024) or youth whose parents also experienced PTS symptoms (Hendriks et al., 2017; Ooms-Evers et al., 2021). As youth with PTS after family violence may also have complex needs, their perceptions and experiences over the course of intensive trauma treatment may inform further testing of this treatment format for this population.

### Present Study

To improve treatment adherence, accelerate recovery, and address the consequences of family violence at the individual and family level, the Child and Youth Trauma Centre in the Netherlands developed intensive trauma treatment with parent and family components. The effectiveness of this intervention, Family-based Intensive Trauma Treatment (FITT), was tested in a randomized controlled trial comparing intensive trauma treatment with parental and family components with intensive trauma treatment without these components (ITT) and with regular non-intensive trauma treatment with parental and family components. Although the results of this study will help us understand whether FITT is an effective treatment for youth exposed to family violence, it is also important to know what worked and did not work from the perspective of youth participating in an intensive trauma treatment with parental and family components (FITT) and those taking part in an intensive trauma treatment without these components (ITT). Therefore we explored youths’ perspectives on improved outcomes and key components of an intensive trauma treatment with and without family and parental components and to also understand what they perceived as facilitators and barriers for treatment success. This qualitative study pursued the following questions:

1. What outcomes did adolescents experience after intensive trauma treatment and how did their perspectives vary with the inclusion of a family-based component?
2. What did adolescents perceive as the key components of intensive trauma treatment and how did their perspectives vary with the inclusion of a family-based component?
3. What facilitators and barriers to treatment success did adolescents experience in trauma treatment?

## Method

### Participants

This study included adolescents with a history of family violence who participated in our randomized controlled trial (RCT) that tested the effectiveness of FITT. The aim of the RCT was to test the effectiveness of parental and systemic components that complement intensive trauma treatment and to test the added value of intensifying trauma treatment. Therefore, FITT was compared with two other trauma treatments: intensive trauma treatment without parental and systemic components (ITT) and non-intensive, weekly trauma treatment as usual (TAU). The RCT is a multicentre research study taking place in four outpatient health care settings for youth in the Netherlands. For detailed information, we refer to the published research protocol (Fictorie et al., 2022). The present study was approved as an amendment to the trial protocol by the Medical Ethical Committee of Amsterdam UMC on 8 June 2023 (reference no: 2020.505). To participate in the initial RCT, participants were found eligible to participate if (a) they had been exposed to family violence, including interparental violence, child abuse and/or sexual abuse; (b) there were no immediate safety concerns anymore; (c) they were between 12 and 20 years old; (d) lived at home with caregiver(s); (e) parents / caregivers, living with the adolescents, could participate in the systemic components; (f) they had symptoms of posttraumatic stress, at least intrusions and avoidance, based on the intake; and if they had (g) fluency in the Dutch language. Adolescents could not participate if they or their caregivers had acute psychotic symptoms, severe alcohol and / or drug addictions, and if adolescents had attempted suicide in the past three months. Only adolescents who participated in FITT or ITT in the RCT could participate in the qualitative study. Nine adolescents participated in the qualitative study. Of them, five received ITT and four received FITT. Treatment took place at one of the four participating treatment centres. Participants in the qualitative study were between 14 and 20 years of age at the time of treatment. One boy and eight girls participated. To protect the privacy of the participants, all participants are called ‘she’ and all names used in this report are pseudonyms. The quotes presented in the result section are verbatim.

Family members who perpetrated family violence in the past were not excluded from the treatment program if family safety was assessed by Child Protections Service, if there was a safety plan, and if there were no immediate safety concerns. When therapists observed or suspected family violence and / or neglect, they were obligated to use the Reporting Code (“Meldcode”)”, a protocol that is mandatory for healthcare professionals in the Netherlands. Part of this protocol is reporting to Child Protection Service in cases of immediate safety concerns or ongoing family violence and / or neglect. Both treatment protocols included information on assessing family safety and establishing a safety plan in cases of continuing violence.

### Procedure

#### Study Procedure

For the RCT, adolescents between the age of 12 and 20 years old, who were referred to one of the participating trauma centres after being exposed to family violence, were assessed for eligibility. In the qualitative study participants were contacted by phone if they had provided consent in the initial study to be contacted for future studies, after the last measurement of the trial. Interested participants received an information letter, explaining that the study focuses on youths’ experiences with the treatment they participated in, including what they perceived as its strengths and weaknesses. Those who agreed to participate signed an informed consent form. In cases were the person was under 16 years of age, parents / legal representatives were asked to sign a consent form as well. After participation, the adolescents received a cinema voucher with a value of 50 euro. 22 participants were contacted, twelve did not want to participate, and one could not be reached. The interview transcripts were de-identified. The first and second author were responsible for coding the interviews and the first author undertook the data analysis.

#### Treatment

Information on the interventions FITT and ITT can be found in Table 1. In both treatments therapist rotation was applied. One therapist coordinated the treatment and also served as a contact person for the participant. FITT is a four and ITT a three week outpatient treatment program. The first week in FITT and ITT consists of two sessions, divided over two days. Week two and three include three treatment days per week, each consisting of three trauma-focused sessions for youth. Because in FITT family members also participate, FITT includes a fourth week with three sessions of family therapy. In FITT the family network also participates in the psycho-education session in week 1. Furthermore, in FITT in week two and three parents receive parenting training on all treatment days and each treatment day ends with a short parent-child interaction session. In total ITT consists of 29 sessions, spread over 11 treatment days. FITT comprises 41 sessions, distributed over 14 treatment days.

**Table 1:** Overview of treatment

Phase	Days *p*/w	Included in	Session	Duration in minutes	Focus	Target
Preparation 1 week	2	FITT & ITT	Psycho-education	90	- knowledge about family violence, PTS, trauma - traumasensitive parenting – rationale of (F)ITT	FITT: adolescent, family (network) ITT: adolescent
		FITT & ITT	Clustering	90	- Ranking traumatic memories	FITT & ITT: adolescent
Trauma-processing 2 weeks	3	FITT & ITT	Imaginal Exposure	90	- PTS and comorbid symptoms	FITT & ITT: adolescent
			Psychomotor therapy	60		
			EMDR	90		
		FITT	Parent training	90	-parental stress - adolescent and parent PTS - support - trauma-sensitive parenting - family narrative	FITT: parents
			Short interaction	15	- sharing - trauma-sensitive parenting	FITT: adolescent and parents
Integration 1 week	3	FITT	Family sessions	90	- trauma narrative - destructive communication patterns - vulnerability cycle - family safety	FITT: family

#### Interview

The interviews were conducted by two clinical psychologists (first and third author) and one postdoc researcher (second author). Both clinical psychologists developed the treatment protocol of (F)ITT but were not involved in the treatment of the participants they interviewed. All interviews were audio recorded and transcribed by two student assistants. The interviews took approximately one hour and, depending on the possibilities and preferences, were done face-to-face at one of the treatment centers (*n* = 7) or online in a secure digital environment (*n* = 2). All participants were informed that if they did not want to answer questions or did not want to complete the interview, this could be done without the need to provide an explanation or experience any consequences. Open exploratory questions were asked about the overall experience with (F)ITT, the perceived outcomes, the proposed key elements, and the perceived facilitators and barriers to treatment success. Verbal interview questions were supported with visual materials. All the different components of (F)ITT were visually illustrated. For example, psychomotor therapy was illustrated with a pictogram showing a stretching exercise. In one of the questions, participants were asked to rank the components according to their level of helpfulness, starting with the most helpful and ending with the least helpful. They were also asked to rank the components according to their level of pleasantness. Examples interview questions: “Which component helped you the most?” and “To what extent did you talk to your parents about treatment?”. In one of the questions participants were asked to rate their overall relationship with the different therapists on a scale from 0 (very negative) to 10 (very positive). The interview protocol is included in the Supplementary Material.

### Data Analysis

Initial codes were defined a priori (Braun & Clarke, 2006), based on the treatment goals/components derived from a literature review focusing on reviews of parenting interventions after family violence, as well as an expert meeting. In this meeting these treatment goals/components were presented. Experts in the scientific and clinical field of family violence, including therapists, social workers and researchers, discussed these goals/components and added additional ones. These goals/components were formulated as codes in the data analysis to explore youths’ perspectives on these goals/components when reflecting on treatment outcomes, key components and facilitators and barriers for treatment success. These defined codes were: (1) trauma-focused therapy, (2) therapist rotation, (3) psychoeducation / knowledge of trauma and family violence, (4) parent-child relationship, (5) parenting, (6) social support / resources, and (7) safety within the family. The first step in analyzing the interviews was for both researchers to read the transcripts. The next step was to read the transcripts again and to make notes for ideas on coding. Then, using the software program ATLAS.ti 25 (ATLAS.ti Scientific Software Development GmbH, 2025), the researchers independently performed general coding by selecting areas of text and assigning these to the respective code. In the next phase, the two researchers reviewed the coded data to determine if the preliminary codes were suitable and, according to inductive coding, if additional codes should be added. The interviews were coded independently, but in joint consultation. Coding choices were discussed to achieve consensus. As the analysis progressed, participants introduced additional concepts, leading to the inductive identification of three further codes. The following codes were added: (1) adverse events, (2) body-oriented therapy, (3) care after treatment.

We determined interview saturation using the method of Guest et al. (2020), a systematic method designed for thematic saturation in qualitative studies. This method calculated the saturation ratio by counting the number of unique themes for every two additional interviews and dividing this by the number of unique themes in the base size. We chose a base size of 4 because most unique themes are collected in the first number of interviews (Guest et al., 2020). After the base size (*n* = 4) three new codes were identified in addition to the seven initial codes, resulting in ten codes. In the subsequent run of two interviews, no new codes were identified. Therefore these two interviews yielded 0% of new information and this was below our new information threshold of 5%. This means that saturation was reached after six interviews. However, we decided to use the data of all nine interviews in the analysis to strengthen the robustness of the results and increase the depth of the answers. The ten codes have been brought together into five overarching themes: (1) family relationships and parenting; (2) trauma treatment; (3) format of the treatment; (4) safety and revictimization; and (5) support outside the family and resources during and after treatment. The themes and codes are shown in Table 2 and then described further in the Result section.

**Table 2:** Themes and codes

Themes	Codes
Family relationships and parenting	Parent-child relationship
	Parenting
Trauma Treatment	Trauma-focused therapy
	Body-oriented therapy
Format of the treatment	Psychoeducation / knowledge of trauma and family violence
	Therapist rotation
Safety and revictimization	Safety within the family
	Adverse events
Support outside the family and resources during and after treatment	Social support / resources
	Care after treatment

## Results

Findings are reported per research question. Within each research question, the results are presented across the identified themes.

### Outcomes

#### Family Relationships and Parenting

After treatment, youth reported both negative and positive outcomes and their perspectives differed, depending on whether they received FITT or ITT. Three participants in ITT felt lonelier, more depressed, or more withdrawn than before treatment. One of them felt that she was doing better and was more open to others after ITT, but conflicts between family members had escalated again and set back their relationships for a long time. Another participant in ITT felt that the relationship with her parents had already improved before start of treatment, because her parents knew what happened to her, offering them a new perspective on the behavior of their child. A participant in FITT, as well as a participant in ITT, felt that her parent also needed a form of trauma therapy. One of them explained that her parent was triggered in situations related to traumatic events they experienced as a family. Unlike the parent in ITT, the parent in FITT was also referred to therapy for herself, to the relief of the participant. All participants in FITT perceived an improvement in the degree to which their parents were supportive. They mentioned that parents were more aware of the effects of trauma and were more understanding than before treatment. As a result, they were more likely to share their feelings with their parents. One participant in FITT felt that her parent was more capable of listening to her and accept her boundaries when she did not want to talk. This increased understanding was articulated by Elena, a participant in FITT, as follows:

*“Because my mom*,* like*,* normally she didn’t really have… um*,* the bond was just very different than it is now*,* you know? She used to get irritated quickly*,* like… by the things I did*,* and she didn’t really have much understanding for it. She didn’t get why I did certain things. She just didn’t understand it. But now*,* she does—she understands it better now.”*

In addition, Lisa, who participated in FITT, described:

This now feels much more like a home.

#### Trauma Treatment

All participants, regardless of whether they received FITT or ITT, were positive about the results of the trauma-focused components with respect to their PTS symptoms. They reported less or no intrusions and nightmares and a decrease in avoidance behavior. They were less afraid in daily situations and during confrontations with objects, persons, or situations that used to be triggers. They experienced more tools to regulate stress, their sleep improved, and they were more aware of body sensations. Lisa said:

*“Well*,* I’m doing pretty well in that I dare to do things*,* and that I dare to go outside*,* you know. And that I dare to live a normal life again.”*

Sofia described:

Well, actually perfect. I haven’t had any problems since.

#### Format of the Treatment

Both FITT and ITT participants reported that during treatment their knowledge increased about the consequences of trauma and family violence as well as of what to expect from treatment.

#### Safety and Revictimization

ITT and FITT participants differed in their perspective on family safety during and after treatment. Both ITT and FITT participants reported a decrease in incidents of family violence. However, three ITT participants reported a continuation of conflicts during and after treatment, although there were no immediate safety concerns, while only one FITT participant reported one incident of violence after treatment. Two ITT participants and two FITT participants reported adverse experiences outside the family in the period after treatment.

#### Support Outside the Family and Resources During and After Treatment

Participants, regardless of whether they participated in ITT or FITT, mentioned that they felt more motivated to do things after finishing treatment, such as exercise or starting a new study. They were less afraid to participate in the normal things of life. They also reported that they were better at taking breaks and talking to others about themselves. Some felt more confident and others experienced more stability in their life, in part because they were better able to regulate their emotions.

### Key Components

#### Family Relationships and Parenting

Participants in ITT and FITT differed in their opinions regarding the necessity of parent and family components, although most indicated they would appreciate their parent receiving support for their own well-being. When asked which component was most helpful, none of the FITT participants mentioned the parent and family component as most helpful. Two out of five ITT participants expressed a preference for a family-based treatment, while the others were pleased that their parents were not involved. Overall, ITT participants assumed that they did not need the parental involvement in treatment because they were used to doing things alone and relied on themselves. Two participants were uncertain whether involving parents would lead to positive change, sometimes due to the parents’ psychological problems. They were afraid that their parent might react with anger, disappointment or lack of self-reflection. One ITT participant was afraid she would not open up if her parents were present, assuming that addressing family relationships would require parents participating in trauma sessions. Two ITT participants were initially pleased that they were assigned to ITT but later felt that they would rather have had FITT. Before treatment, one feared parental anger upon disclosure, while the other preferred to handle matters alone. Subsequently, both felt that support for their parents could have improved parental understanding and support and would have benefited the parents themselves.

One FITT participant assumed that the outcomes would have been similar even if parents had not been involved, although she valued the support she received during the parent-child interaction sessions. The other three FITT participants were pleased to have received family-based treatment, attributing this to improved outcomes on a family level. Youth participating in FITT reported that it was important for them to see that their parent receive help and support. Another participant in FITT noted that FITT created a context in which the parent could talk about the subject of family violence, which was considered to be important because the parent had also experienced family violence in childhood. Another FITT participant felt that after FITT her parent was more sensitive and this made it easier for her to talk to her parent. Lina, who participated in FITT, valued the interaction session at the end of each treatment day:

*“I could still find my support at the end of the day. Because everything had been so intense and then at the end of the day you still had that support. That you could still talk to her.”*

#### Trauma Treatment

Participants were asked which component they valued the most, including Imaginal Exposure, PMT, and EMDR. No differences were found between ITT and FITT participants. Three participants were most positive about Imaginal Exposure therapy. Sofia said:

*“I just couldn’t get the words out. Just saying the words. At some point*,* I managed to do it*,* and I really feel like that helped the most.”*

Although five participants reported Imaginal Exposure as the therapy they disliked the most, they experienced it as very successful in reducing their PTS symptoms. Elena described:

*“I found it awful to do*,* but yeah*,* in some way I also kind of valued it… like… to come back into the moment and realize: yes*,* this is what happened. And then*,* in the end*,* I can just let it go.”*

Four participants experienced EMDR as the most helpful part of the program, while there was also a participant who disliked EMDR and felt that it was not effective. PMT was mentioned three times as the most effective part, and almost all participants appreciated PMT and reported that it increased their body awareness and offered them tools for stress regulation. One reported that PMT was uncomfortable. A participant who described always feeling tensed was surprised that she could feel a moment of relaxation during PMT. In addition, a participant mentioned that PMT was the fun part of the day and valued the information she received about the effects of trauma on the body. Participants in general appreciated the psychoeducation during PMT and the opportunity to release stress.

#### Format of the Treatment

No differences were apparent between the perspectives of FITT and ITT participants regarding key components. Participants described the combination and coherence of the various trauma therapies as key components. Layla said:

*“So you have the part where you really go into your head*,* you have the part where you’re actively doing something but still thinking about it and kind of processing it*,* and then you have physical therapy*,* which worked really well for me. Because for me*,* I work better in practice—books are really difficult for me. And through that*,* we actually discovered that this approach worked really well.”*

Participants valued the variety of trauma-focused therapies included in the treatment, as this ensured that there was always a therapy that suited their individual needs. Clear explanations regarding the rationale behind the trauma therapies further enhanced their positive expectations of the therapy. Participants reported that the psychoeducation session provided comprehensive information about the treatment program, and valued that the same information was repeated during treatment. Sofia said:

I knew what I could expect and what I expected also happened.

All participants looked back positively on the support they received by the team of therapists during the treatment. On average, participants gave the team an 8.3 on a scale ranging from 0 to 10 for the therapeutic relationship. Grades ranged from 6 to 10. Overall, they felt supported and the diversity in the way therapeutic techniques were applied was helpful. One participant mentioned that she would feel more comfortable with a single therapist. Others felt that receiving treatment from multiple therapists prevented them from bonding with a therapist, avoiding sadness when that relationship would end at the end of treatment. In general, participants felt that they could talk about everything with the therapists and that the therapist knew what they were doing. Participants appreciated they had one person who coordinated the whole treatment process.

#### Safety and Revictimization

ITT participants attributed the decrease in incidents of family violence to newly acquired skills on the individual level. For example, an ITT participant experienced fewer fights at home because she was now more inclined to ask what was going on before reacting on an impulse. Another participant felt that after ITT she was better able to cope with specific behavior of her parents that would previously lead to a lot of tension. A third participant in ITT described that she previously had conflicts with her parents due to her oppositional behavior, but since she opened up to them about her traumatic experiences, they were understanding and the number of conflicts decreased. An ITT participant who described conflicts at home, mentioned that after treatment she and her parent did not know how to interact with each other to prevent conflicts. She also missed aftercare on this topic. Layla, another ITT participant, described:

*“I could calmly go home*,* but once you are home*,* you fall into a certain automatic rhythm. You have put on a certain mask.”*

A FITT participant said that her family no longer had physical fights, while they had these prior to treatment, and attributed this to a change in the behavior of all family members. One FITT participant, Lisa, who valued the interaction sessions at each treatment day in week 2 and 3 the most, described:

*“The conversations at the end of the day*,* together with my mother*,* I really enjoyed. I immediately noticed how much I liked that. Because I could*,* in a safe environment*,* talk to my mother without us*,* like yelling at each other. I felt really safe to open up about it. I could see that it was good for my mother.”*

#### Support Outside the Family and Resources During and After Treatment

In general, participants felt that the trauma components helped them feel less tension and fear, resulting in a resumption of normal life. For example, these components helped them to talk and hang out more with friends, be more active at school, and do sports.

### Facilitators and Barriers

#### Family Relationships and Parenting

In the treatment of ITT participants parents were not involved. ITT participants did have fears and assumptions about participation of parents that could serve as barriers for treatment success. An ITT participant thought she would felt afraid to open up with parents present because the parents did not know details of the traumatic experiences, while another ITT participant felt that she would be unable to share traumatic events because her parent was part of the traumatic experiences. Four ITT participants mentioned that parents were a support for them during treatment and this helped them continue treatment. For example, parents drove them to treatment or accompanied them during breaks on a treatment day, were interested in their feelings after a treatment day, or offered them drinks and hugs. One FITT participant explained that trauma triggers prevented her parent to talk about her trauma history.

#### Trauma Treatment

Participants mentioned that it was important to “*just surrender to it”.* Furthermore, it was mentioned that some stability and support in life increased their willingness to engage in trauma therapy. Layla said:

*“I was extremely tense*,* I really thought*,* ‘this is going to be intense*,* this is scary.’ But actually*,* um*,* I received so much support in that moment that it became much easier to approach it calmly.”*

#### Format of the Treatment

Some participants were nervous about how they would respond to the intensity of the program. One participant mentioned feeling scared when anticipating a session in which she had to process a memory that she did not want to think about again. Another participant said that it was helpful that all the therapists were familiar with her life history as this eliminated the need to repeat their story to each therapist. Two participants described the transition from intensive treatment to a treatment pause afterward as a large step and expressed that they missed direct aftercare. They suggested implementing a more gradual transition from high-intensive to low frequency treatment.

#### Safety and Revictimization

One ITT participant mentioned that her home situation was more stable, and this helped her to enter into treatment. One FITT participant reported that it would not have been possible for her to process the traumatic memories if one of her parents, no longer living with the family, was still such a threat. One participant noticed that during ITT, her parent was setting some clear rules and boundaries at home to create a safer and more stable environment. In contrast, another participant experienced a lot of stress at home during ITT. Layla described:

Because before you start the day, you had to fill out a form about how you’re feeling, how things went, and there was always kind of a fixed pattern because of what was happening at home. And then I had to say again that there had been a fight. And I think that was probably on that list every single day.

Three participants in ITT and two participants in FITT mentioned different adverse events in and outside the family after treatment that hindered their recovery, including family separation, bullying, attempted suicide of a loved one, sexual abuse, street violence and recurring family conflicts.

#### Support Outside the Family and Resources During and After Treatment

Participants differed in whether they were open at school or work about their treatment and if they were open, whether they experienced support. For some, informing school and work about their treatment program helped them during and after treatment. They felt supported if school or work was flexible with their schedule and expectations and if colleagues and teachers were emotionally available. A participant reported that she did not talk about her treatment at work and preferred to continue her work during treatment. Some participants also preferred to continue their school on non-treatment days, while others who continued their schoolwork felt it was too much, especially because they experienced pressure from school. Another participant felt that her colleagues were gossiping about her trauma and did not feel supported. Other participants who were open to work or school about their treatment did not have to go to school or work and this felt supportive. One participant described that this resulted in a calm state when entering treatment.

Support from friends was important for participants, although some also felt too tired to meet with friends after a treatment day or felt very lonely during treatment and missed supportive friends. Participants who received support from friends described that they had contact with friends after a treatment day, but friends also accompanied them during the breaks on treatment days. Especially friends who also experienced traumatic events were described as more understanding. Regarding resources, participants were very clear on the positive mindset one needs when starting an intensive treatment. It was important to open up for treatment and to feel motivated because the treatment requires hard work.

## Discussion

The aim of this study was to explore the perspectives of youth on the outcomes and key components of an intensive trauma treatment with and without a family-based component and their perceived facilitators and barriers to treatment success. Their answers could be clustered within the following five themes: (1) family relationships and parenting; (2) trauma treatment; (3) format of the treatment; (4) safety and revictimization; and (5) support outside the family and resources during and after treatment. From the perspective of youth, the treatment decreased symptoms of PTS, improved regulation skills, increased motivation and confidence to participate in daily activities, improved youth’s understanding of trauma and the consequences of family violence, with the family component especially improving support and understanding of parents, reducing incidents of family violence, and resulting in professional support for parents themselves. Youth who did not receive the family-based component also reported negative outcomes, such as feeling lonely, depressed and withdrawn.

Key components, perceived by youth, were the trauma-focused therapies, including imaginal exposure, EMDR and psychomotor therapy, and the variety of these therapies, the psychoeducation on trauma (treatment) and family violence, and the diversity in therapists’ approach and the supportive therapist team, with the family-based component creating a safe context in which they received support from the parent and could talk about important topics. Most youth felt that support and treatment for parents themselves were or, if they did not receive the family-based component, could be key components. Some youth receiving intensive trauma treatment without a family-based component assumed this component would not have added value. They expressed that they were accustomed to relying on themselves and were doubtful if parents would benefit from such a component, or wary of their parents’ reactions.

Perceived facilitators for treatment success were some stability in daily life, an open attitude and motivation toward treatment, and support from parents, school, work, and peers, especially those peers who had experienced traumatic experiences themselves. Barriers to treatment success were ongoing threat and family violence, fear of the trauma-focused components and abrupt transition from intensive to low-frequency treatment or a treatment pause. For youth participating in intensive trauma treatment without a family-based component, the assumption that details of the traumatic memories needed to be shared was also a barrier, as well as fear of including parents who were associated with the traumatic memories.

The findings not only provided insights into youths’ views on treatment outcomes, key components and factors facilitating or barring treatment success, but also offer hypotheses on how youth explain change throughout their therapeutic process. They perceived that clear expectations of the treatment helped them to endeavor and pursue the trauma-focused components. They perceived that psychoeducation increased their knowledge of trauma and family violence and that this offered an explanation for their problems and a rationale for the treatment plan. This is in line with a previous study that showed the importance of psychoeducation in decreasing PTS symptoms in adults exposed to family violence (Laun, 2015). For a treatment to be effective, it is important to have faith in the explanation of the psychological problems and in the rationale and actions of the treatment (Wampold, 2015). This was also shown by the comments of participants on Imaginal Exposure Therapy. They disliked this form of therapy but understood why it was important to engage in it, and afterwards perceived it as having been very effective. Their understanding of the rationale of the therapy implies that they agreed on the therapy goals and tasks, elements of the therapeutic alliance, and this alliance leads both to more positive expectations of the treatment and a better therapeutic relationship (Crits-Christoph et al., 2006). Youth were very positive about the therapeutic relationship with the therapist team. They felt supported by the therapists and described how the diversity of the therapist’s approach helped them benefit from the combination of trauma-focused components of the treatment. Some felt that therapist rotation prevented them from becoming sad about parting with a therapist whom they might have bonded with. This was a remarkable finding and sheds new light on the term therapeutic relationship. It resembles the results of a qualitative study on intensive trauma treatment with adults (Thoresen et al., 2022) in which participants described that therapist rotation prevented them from attaching to one person during therapy and offered space to focus on their own process in the specific factors of the treatment.

The study findings, based on the perspectives of youth, suggest that the addition of a family-based component possibly enhances treatment for youth exposed to family violence. Youth receiving the family-based component perceived that the family-based components improved parents’ support and understanding and created a safe context to talk to parents about difficult matters. Youth who did not receive the family-based component also felt that the presence of parental support helped them during treatment. This is in line with a systematic review in which emotional availability was found to be a moderator between family violence and children’s internalizing symptoms (Carter et al., 2022). It could be that a supportive and understanding context could help youth pursue treatment and benefit from the specific components of a treatment. However, youth who did not receive a family-based component also described how changes at the individual level, such as increased coping skills and reductions in PTS, indirectly reduced the risk of family conflicts. Furthermore, despite family conflicts in several families, all participants reported positive outcomes regarding their PTS symptoms. Perhaps youth can benefit from treatment, despite ongoing conflicts. This is in line with a study of Lünnemann et al. (2022) who found that levels of PTS symptoms decreased when the number of family conflicts diminished but these conflict did not totally stopped. However, participants also reported that continuing threat could form a barrier, where some stability in life was seen as a facilitator of treatment success. Some participants who did not receive the family-based component also reported negative feelings after treatment, such as loneliness. It should also be noted that new adverse events after treatment, can result in a recurrence of symptoms.

The study findings also showed that the format of the treatment could play a role in enhancing treatment. Youth valued the treatment format, including psychoeducation on trauma and family violence, a clear rationale of the treatment plan, a variety and combination of trauma-focused components, and the use of therapist rotation. Comparable to the findings of the meta-analysis of Hoppen et al. (2023), it could be that this format helped them complete treatment. Youth perceived that the combination and variety of the trauma-focused components, including Imaginal Exposure, EMDR and Psychomotor therapy, elicit actions that reduced PTS symptoms and improved stress regulation and coping skills. This corresponds to the views of therapists, who identified the multiple trauma-focused components as a key element of intensive trauma treatment (Vaage-Kowalzik et al., 2025).

### Limitations and Strengths

An important limitation of this study is that, due to the small number of participants, variation within the two groups was limited. In addition, the risk of sampling bias means that this study describes the experiences of a specific group of participating youth. A second limitation is that the time interval between the end of treatment and the interview varied and it could be that retrospective reflections vary depending on this time interval. An important strength of this study is that we succeeded to collect detailed information on the perspectives of youth who are often excluded from research because of safety concerns. Also, the findings challenge the prevailing assumption that trauma treatment can only commence once family violence has fully ceased.

### Implications

Although participants in FITT valued the family-based component, the presumptions of participants in ITT towards this component were mixed and therefore it is important to carefully address their concerns and the possible negative beliefs they have about including their family in treatment. Clear information on the content of a family-based component is necessary and shared decision-making (Langer & Jensen-Doss, 2018) can help to include both youth and parent perspectives. A second recommendation is to include intensive trauma treatment as one of the treatment options, as this form of treatment is valued by youth. When offering intensive trauma treatment, it is advisable to plan how the transition from intensive treatment to non-intensive treatment or guidance can meet the needs of youth and parents. Another recommendation is to ensure that youth who experience ongoing family conflicts are not automatically excluded from treatment as several participants in this study profited from treatment, despite ongoing conflicts at home. A last recommendation is to consider how support outside the family can be integrated in the treatment, for example, by involving a peer from their own network, other adolescents who participate in the same treatment program, or by including a peer expert. Although the effect of peer experts in the treatment of youth has not yet been established (Simmons et al., 2023), youth in the present study valued support from peers with a trauma history and youth in the study by van Ee et al. (2024) explained that social support, in the form of acknowledged feelings, distraction and the possibility of sharing feelings, was important for them to start and adhere to therapy.

### Conclusion

Youth exposed to family violence reported valuable outcomes after intensive trauma treatment with and without a family-based component. It is promising that this group, which is sometimes withheld from trauma treatment because of unsafe home situations or parent PTS, reported improvements, even in cases where family conflicts continued. Both the format of the treatment and the family-based component were mentioned as key components. It is important to carefully address the beliefs of youth on participation of families in trauma treatment and follow a process of shared-decision making. Support in general can help youth to profit from intensive trauma treatment. To enhance trauma treatment for youth, systematic evaluations of group trials are necessary to determine whether family-based components can be recommended.

## Data Availability

Because of privacy regulation and data protection, the participant-level dataset is not available.

## Declarations

### Clinical Trial Registration

The original trial was registered in the Netherlands Trial Register (TrialNL8592) on 4 May 2020. The protocol was published in 2022.
